# Fresh Fish Degradation and Advances in Preservation Using Physical Emerging Technologies

**DOI:** 10.3390/foods10040780

**Published:** 2021-04-05

**Authors:** Jéssica Tavares, Ana Martins, Liliana G. Fidalgo, Vasco Lima, Renata A. Amaral, Carlos A. Pinto, Ana M. Silva, Jorge A. Saraiva

**Affiliations:** 1 LAQV-REQUIMTE, Department of Chemistry, University of Aveiro, 3810-193 Aveiro, Portugal; jessica.tavares19@ua.pt (J.T.); ana.patricia.martins@ua.pt (A.M.); liliana.fidalgo@ipbeja.pt (L.G.F.); vasco.lima@ua.pt (V.L.); renata.amaral@ua.pt (R.A.A.); carlospinto@ua.pt (C.A.P.); 2 Department of Applied Technologies and Sciences, School of Agriculture, Polytechnic Institute of Beja, 7800-295 Beja, Portugal; 3 SONAE, Lugar do Espido, Via Norte, 4471-909 Maia, Portugal; amsilva@sonaemc.com

**Keywords:** fresh fish, spoilage, shelf-life, chilling/refrigeration, freezing, edible coatings, hyperbaric storage

## Abstract

Fresh fish is a highly perishable food characterized by a short shelf-life, and for this reason, it must be properly handled and stored to slow down its deterioration and to ensure microbial safety and marketable shelf-life. Modern consumers seek fresh-like, minimally processed foods due to the raising concerns regarding the use of preservatives in foods, as is the case of fresh fish. Given this, emergent preservation techniques are being evaluated as a complement or even replacement of conventional preservation methodologies, to assure food safety and extend shelf-life without compromising food safety. This paper reviews the main mechanisms responsible for fish spoilage and the use of conventional physical methodologies to preserve fresh fish, encompassing the main effects of each methodology on microbiological and chemical quality aspects of this highly perishable food. In this sense, conventional storage procedures (refrigeration and freezing) are counterpointed with more recent cold-based storage methodologies, namely chilling and superchilling. In addition, the use of novel food packaging methodologies (edible films and coatings) is also presented and discussed, along with a new storage methodology, hyperbaric storage, that states storage pressure control to hurdle microbial development and slow down organoleptic decay at subzero, refrigeration, and room temperatures.

## 1. Introduction

Fish is a highly demanded and nutritious food product, yet perishability remains the biggest challenge for its preservation [1]. This food must be stored refrigerated or frozen, and, even under those conditions, it has a very short shelf-life, particularly for refrigeration (5–7 days and 9–12 months under refrigeration and frozen conditions, respectively) [2]. The deterioration of fresh fish during storage is attributed to different damage mechanisms, like microbiological spoilage, autolytic degradation, and lipid oxidation [3].

Fish products contain important nutritional and digestive proteins, including essential amino acids, lipid soluble vitamins, micronutrients, and highly unsaturated fatty acids. The muscle is mostly composed of water (75–85%), and it has a high water activity (0.98–0.99) [4]. Protein represents 20–22% of the muscle [5], while many types of lipids with different chemical composition, such as neutral/non-polar (triglycerides, diglycerides, etc.) and polar (free fatty acids, phospholipids, etc.) lipids, are also present [6]. Fish can be divided in four basic groups regarding its fat content: lean (<2% fat), low-fat (2–4% fat), medium-fat (4–8% fat), and high-fat (>8% fat) [7].

### 1.1. Fish Spoilage

After fish are caught, spoilage starts rapidly, and rigor mortis is responsible for changes in the fish after its death. A breakdown of various components and the formation of new compounds are responsible for the alterations in odor, flavor, and texture that happen throughout the spoilage process, and deterioration occurs very quickly due to various mechanisms triggered by the metabolic activity of microorganisms, endogenous enzymatic activity (autolysis), and by the chemical oxidation of lipids [1,8].

#### 1.1.1. Autolytic Enzymatic Spoilage

Initially, the main autolytic changes happening are the enzymatic degradation of adenosine 5′-triphosphate (ATP) and its related products, followed by the action of proteolytic enzymes [9], as reported in Table 1. The concentrations of ATP and its breakdown products (adenosine 5′-diphosphate (ADP), adenosine 5′-monophosphate (AMP), inosine 5′-monophosphate (IMP), inosine (INO), and hypoxanthine (Hx)) are one of the most effective and reliable indicators of fish freshness (K-value), varying according to the fish species, muscle types, and storage conditions [1,9]. The K-value, which increases with spoilage, is calculated according to the following ratio (Equation (1)):

(1) $$K-\mathrm{value}\;\left(\%\right)=\frac{100\times \left(\mathrm{INO}+\mathrm{Hx}\right)}{\mathrm{ATP}+\mathrm{ADP}+\mathrm{AMP}+\mathrm{IMP}+\mathrm{Hx}}$$

High autolytic activity of the major muscle endogenous proteases causes the hydrolysis of key myofibrillar proteins, contributing to the weakening of the myofibril structure during post-mortem storage. The main proteolytic systems in place are the cytoplasmic calpains (at neutral pH) and the lysosomal cathepsins (at acid pH), such as cathepsins B, L, H, and D [11].

**Figure 1:**
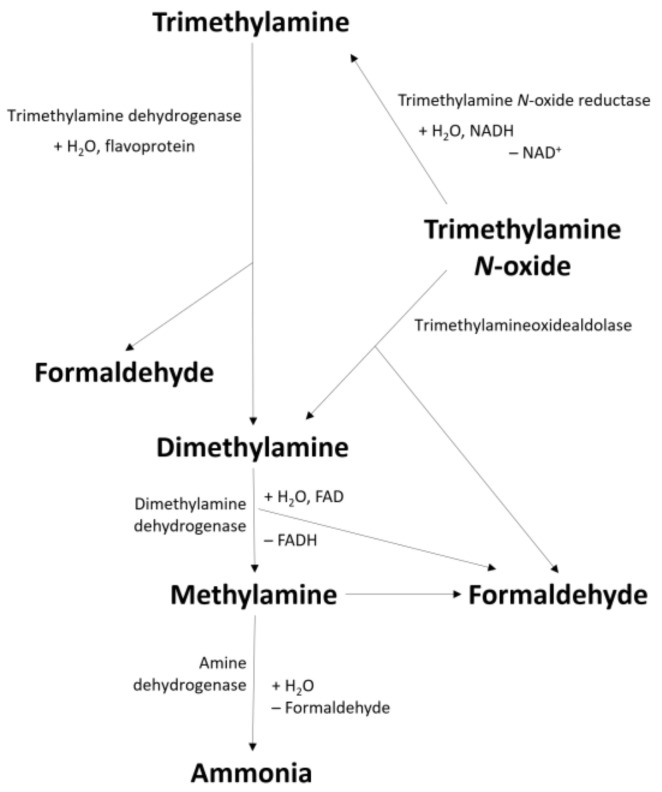
Degradation of trimethylamine and its N-oxide compounds [5].

Trimethylamine (TMA) and its N-oxide compounds are usually used as indices for freshness in fishery products. The pathway of the production of formaldehyde and ammonia from TMA and its N-oxide is shown in Figure 1, which are associated with the formation of undesirable odors, and occur in fish by the action of several enzymes, such as trimethylamine N-oxide reductase (TMAO reductase), trimethylamine dehydrogenase (TMA dehydrogenase), dimethylamine dehydrogenase (DMA dehydrogenase), and amine dehydrogenase [5]. Total volatile base nitrogen (TVB-N) and trimethylamine-nitrogen (TMA-N) are quality indicators traditionally used for fish products [12]. TVB-N includes the measurement of volatile basic nitrogenous compounds associated with seafood spoilage, like TMA produced by bacteria, DMA derived from autolytic enzymes action, ammonia produced by the deamination of amino acids, and others [13]. A value of 35 mg N/100 g is proposed as the upper limit for the spoilage initiation [13]. However, some studies present lower limits, depending on the results obtained and the studied fish species [13,14,15]. TMA-N typically has a fishy odor, and it is produced by the decomposition of TMA N-oxide (major constituent of non-protein nitrogen fraction) caused by bacterial spoilage and enzymatic activity. The upper limit of acceptability is typically around 10–15 mg TMA-N/100 g, however, like TVB-N, lower limits are suggested by other authors [12,13,14].

#### 1.1.2. Lipid Hydrolysis and Oxidative Spoilage

Fish quality can also be affected during storage at different temperatures by lipid oxidation through odors and lipid peroxide formation or by taste, texture, consistency, and nutritional value losses. Transition metals are primary activators of molecular oxygen, leading to oxidation, which consists of oxygen reacting with the double bonds of fatty acids, mostly of polyunsaturated fatty acids (PUFAs), that are highly susceptible to oxidation [16]. Lipid oxidation can occur either enzymatically or non-enzymatically in fish. In the enzymatic hydrolysis (lipolysis) process, glycerides are split by lipases, forming free fatty acids that are responsible for the common off-flavor (rancidity) and from the denaturation of sarcoplasmic and myofibrillar proteins [17]. Main lipolytic enzymes include triacyl lipase, phospholipase A2, and phospholipase B [3], and they can either be endogenous or derived from psychrotrophic microorganisms [17]. Furthermore, the presence of pro-oxidant enzymes, like lipoxygenases and peroxidases, facilitates lipid oxidation [18].

Non-enzymatic oxidation is triggered by the catalysis of hematin compounds, such as hemoglobin, myoglobin, and cytochrome, generating hydroperoxides. The peroxides are unstable and susceptible to hydrolysis, forming volatile compounds (like aldehydes, ketones, and alcohols), which causes off-flavors [19].

Lipid oxidation is relevant for fish quality due to the development of off-odors, especially in fatty fishes. Normally, the degree of lipid oxidation is given by a thiobarbituric acid (TBA) value that measures the malondialdehyde (MDA) content that is formed by the reaction with hydroperoxides (initial products of lipid oxidation). TBA values of 2–4 mg MDA/kg are within quality limits [13]. Nevertheless, this value might not reflect the real rate of lipid oxidation, because MDA can interact with other components of the fish body and produce secondary metabolites that include reactions with carbohydrates, furfural, alkenals, alkadienals, and other aldehydes and ketones [13].

So, in fish, lipid oxidation consists of a complex chain of reactions, with three distinct phases: primary (formation of hydroperoxides), secondary (e.g., hexanal and malondialdehyde formation), and tertiary/interaction compounds (new compounds are formed by the breakdown of secondary oxidation products or through the reaction with other molecules, mostly nucleophilic type) [20].

#### 1.1.3. Microbial Spoilage

Microbial growth is the first mechanism deteriorating fish, being the spoilage factor that most affects the quality of fresh or lightly preserved fish [9]. Initially, the fish muscles are sterile, but after death, they are contaminated by the microbial population present at the fish skin [21]. The high water activity, low acidity (pH > 6), and high amount of non-protein nitrogenous compounds typical of fish results in the fast growth of microorganisms, leading to undesirable changes in appearance, texture, flavor, and odor, reducing its quality. Spoilage created by microorganisms generates volatile amines, biogenic amines, organic acids, sulfides, alcohols, aldehydes, and ketones, which have unpleasant and unacceptable off-flavors [22]. The main compounds formed during microbiological spoilage are listed in Table 2. Biogenic amines, such as histamine, cadaverine, tyramine, and putrescine, are produced by the decarboxylation of specific free amino acids by microorganisms during storage, and are used to monitor fish safety and quality [23,24].

The ability to produce histamine is known in different species of bacteria that have histidine decarboxylase [27]. Being extremely stable, histamine cannot be easily removed or destroyed by cooking, retorting, or freezing [28], and, among amines, it is toxicologically relevant, causing scombroid fish poisoning and food intolerance [29].

It should also be noted that the existing physicochemical conditions and the interactions between the microorganisms will impose a selection of the organisms capable of growing under such conditions. The initial microflora of the fish is dependent on different factors, such as the environment where the fish lives, the fishing season, water temperature, the method of capture, the handling on the ship, or the technological and sale process [21], but, regardless of the variety of microflora present in the fresh fish and the diverse parameters used for preserving, the species growing are consistent in the different products. From the different microbial species that can develop on fish, only one or a few will produce the off-odors and off-flavors, named specific spoilage organisms [30].

## 2. Chilling, Superchilling, and Freezing

The preservation of food products without using preservatives or additives has been increasingly demanded among consumers, and has brought additional challenges, especially to highly perishable foods, such as meat or fish. Low temperatures during the capture, transportation, and storage of the fish are of major importance worldwide. Chilling, superchilling, and freezing techniques allow for the preservation of fish for longer periods without major changes in quality, and assure economic benefits for the fish companies [31]. Therefore, chilling is one of the most used methods for fish preservation, along with freezing and, recently, superchilling.

### 2.1. Chilling

Chilling is the process of cooling fish or fish products to a temperature approaching that of melting ice, using, for example, ice. Chilling promotes an increase of shelf-life by slowing physical and chemical reactions and the action of deteriorative microorganisms and enzymes [31,32].

Chilled fish can keep a high organoleptic quality, being highly attractive for consumers, however, it is susceptible to microbial safety problems due to the temperature range at which it is kept, since psychrotrophic pathogens can grow and proliferate without an obvious sensorial impact [33].

Usually done with ice, chilling can maintain the fish at temperatures close to 0 °C and extend the shelf-life up to 30 days (in fatty fish, this can be up to 40 days), depending on several factors, such as the water temperature (temperate or tropical waters) and the type of species (marine or freshwater species) [31]. The shelf-life of different fish species stored in ice in shown in Table 3. However, temperatures close to 0 °C are not easily possible at retail and consumer houses, and, therefore, refrigeration (storage above 0 °C and up to 5 °C), the most usual storage process for fresh fish, results in a much shorter shelf-life [34].

### 2.2. Superchilling

Superchilling, also known as partial freezing or deep chilling, is characterized by low temperatures (between conventional chilling and freezing), in which a decrease of 1–2 °C occurs below the initial freezing point of the food product [32,36,37]. Most foods have a freezing point that varies from −0.5 to −2.4 °C and, specifically for fishery products, this parameter is between −0.8 and −1.4 °C [38,39].

The superchilling process results in the conversion of a small fraction of water (≈5–30%) into ice, forming a thin layer of ice (≈1–3 mm) on the surface of the food and an internal ice reservoir [40,41]. Thus, the combined effect of low temperature and internal/external ice on food produce slows deteriorative processes (such as microbial activity) and, for short periods, ice may not be necessary during transport or storage [32,36,40,42]. 

Ideally, in superchilling, a small amount of water content is transformed into ice and therefore, there is less freeze protein and structural damage (detachments and breaks of myofibers) by ice crystals compared to frozen storage. The shelf-life of superchilled food can be one and a half to four times longer when compared to the chilling process due to the reduction of microbial and enzymatic activity [32,37,39,41]. For example, according to [43], the shelf-life of fresh cod loins (*Gadus morhua*) has been extended from nine days in ice-chilled storage to 16 or 17 days in superchilled storage, which means that the shelf-life has increased by about 1.9 times. At superchilling temperatures, biochemical/physicochemical reactions are affected (at a higher rate or even accelerated over time), and there are some negative effects on quality parameters, for instance, changes in muscle texture [32,36,39]. Therefore, superchilled technology can be combined with other preservation methods, such as modified atmosphere packaging (MAP) and vacuum packaging (VP) as combined methodologies, to minimize possible detrimental effects [36]. The synergetic effect of MAP and superchilled technologies was described by [44], in which the extension of the shelf-life of fresh Atlantic salmon fillets (*Salmo salar*) was investigated. It was concluded that the salmon samples packaged in a MAP atmosphere (90% CO_2_ and a gas-to-product volume ratio of 2.5) increased from 11 to 22 days in terms of shelf-life when compared to the control samples (wrapped and exposed to atmospheric oxygen). Additionally, the sensorial and physicochemical properties (drip loss, pH, total volatiles amines, etc.) were evaluated, and all were within the acceptable limits, therefore, the shelf-life was determined only by microbial growth [44]. 

Superchilling has raised interest in its application to some food products, namely fishery products, due to the shelf-life extension and quality improvement, in comparison to traditional preservation methods. Table 4 presents the conditions for different superchilled fish species, including data from other preservation technologies and from combination with diverse packaging methods. Therefore, in general, the shelf-life is longer when superchilling technology is combined either with VP or MAP methods and when compared to the shelf-life obtained in each of these individually.

Considering the information presented, it is necessary to understand how the technology influences the degree of superchilling, the growth of ice crystals, and the rate of biochemical reactions (like protein denaturation, enzymatic activity, or lipid oxidation) that indirectly influences quality parameters (like color, texture, flavor, drip/liquid loss, among others) of fish and fishery products [32,36,37,38]. 

Superchilling is also a promising and eco-friendlier technology due to an 18% reduction in environmental impact when compared to the conventional cold chain. Additionally, it improves the overall quality of food and extends its shelf-life by reducing microbiological contamination/propagation and, on an industrial scale, promotes higher production yields and reduces labor and transportation costs [32,42].

### 2.3. Freezing

Of all of the low-temperature preservation methods used, freezing (frozen storage) is the one that can maintain fish and fish products conserved for longer periods, but some quality parameters can be affected. It is typically applied at temperatures between −18 to −40 °C depending on the type of fish stored, and, contrary to what happens with chilling, for frozen storage, most deteriorative and pathogenic microorganisms are unable to proliferate at temperatures below −10 °C [32,52]. At this temperature, approximately 80% of the water is converted to ice, decreasing the water activity, which inhibits microbial activity [53,54]. 

The shelf-life of the frozen fish depends on several factors, such as the initial quality, storage conditions, and fish species, while the quality depends mainly on the storage temperature and temperature fluctuations [52,55]. Table 5 presents the shelf-life of some fish species stored at different freezing temperatures, according to fat content and fish size and shape. Notwithstanding the advantage over chilling regarding the inhibition of microbial growth, the impact of freezing temperatures in quality parameters is quite important when choosing the preservation technique. Some textural changes take place due to the formation of ice crystals that damage the tissues (mainly related to protein denaturation), which promotes dryness and toughness, and occurs more frequently in lean fish than in fatty or semi-fatty fish species. This can be minimized by fast-freezing processes, leading to smaller ice crystals and lower cell wall rupture and drip loss during the thawing process [52,53]. 

Flavor and odor changes also occur in frozen fish due to fatty acid oxidation and development of rancidity [53,57]. Color changes, especially in fatty fish, are directly related to lipid-protein cross links promoting the decrease in protein solubility. These reactions can be minimized by glazing and packaging, and through the exclusion of oxygen and light. In fish like salmon, due to the presence of carotenoids, oxidation occurrence promotes color changes [53,55]. 

The negative impacts of freezing in the quality parameters of the fish can be attenuated by adjusting and controlling storage temperature, rate of freezing, and fluctuation of temperature during storage through several types of freezing processes. Three basic methods can be used for fish or fish products: air blast freezers, contact or plate freezers, and immersion or spray freezers [56,58].

## 3. Emergent Preservation Techniques

Several strategies have been evaluated to increase the shelf-life of fresh fish with minimal impact on quality, particularly texture, and to extend shelf-life compared to refrigeration to try to avoid freezing preservation. These strategies rely on the application of additional hurdles prior to conventional storage, such as edible films and coatings, or even on the application of nonthermal preservation methodologies, such as hyperbaric storage, to slow down microbial proliferation, as in refrigeration, and also to possibly reduce microbial loads to more desirable levels and lessen degradation reactions to increase the shelf-life of fresh fish. This section will cover some of the main emergent approaches to preserve fresh fish, encompassing the main effects of each methodology on microbiological and chemical aspects of fresh fish [59].

### 3.1. Edible Films and Coatings

Edible films and coatings are other innovative strategies applied to food preservation that have been shown to be effective in protecting the sensorial and nutritional properties of food, while improving its safety and prolonging shelf-life by reducing/inhibiting microbial growth during the supply chain [60,61]. In fact, these technologies are similar to active packaging, however, they do not act as a package itself, even though the film/coating is in close contact with the food [62].

Edible films and coatings are defined as a thin layer of edible material, whereby, in the first phase, the film is produced separately (like solid sheets) and placed on the surface or between the food products (as wraps or separation layers, respectively). Meanwhile, edible coatings are formed directly on the surface of the food products by dipping (most used in fish and fishery products), spraying, or a fluidized bed, which are selected according to the characteristics of the food product and the film/coating [41,60,63,64]. 

These films and coatings are bio-based materials, and they are therefore named biopolymers due to their sustainable and eco-friendly source, as residues from the food industry and undervalued components of proteins (such as corn zein, gelatin, and casein), lipids (like shellac resin, waxes, and triglycerides), polysaccharides (such as starch, chitosan, and carrageenan), or their combinations [41,60,61,64].

In addition to high biodegradability, these biopolymers are edible, or can be washed or disintegrated due to further processing [41,65]. The most common natural polymers applied in fishery products are chitosan, alginate, whey proteins, gelatin, or their combinations [41]. Chitosan belongs to the group of polysaccharides, being one of the most abundant, and it has been investigated to be applied as a film/coating material for fishery products due to its non-toxicity, biocompatibility, biodegradability, biofunctionality, antimicrobial and antifungal properties, film-forming properties, selective gas permeability, and low-fat diffusion. Moreover, other protein and polysaccharide biopolymers have been developed for fish and fish-based products [41,63,66].

Similarly to active packaging films, active compounds, such as antioxidants, antimicrobials, and/or flavorants (namely essential oils, natural extracts from herbs and spices, enzymes, and protein hydrolysates, among others) as edible films and coating materials can be added to improve the safety, quality, and stability of foodstuff due to the low/reduced biological activity of biopolymers against spoilage microorganisms. Additionally, other additives, like plasticizers, and crosslinking agents (to improve or modify the physicochemical properties of films/coating polymers) are also incorporated [41,60,61,64].

Edible films and coatings, both biopolymers and active compounds, must comply with European Union Regulations, namely Commission Regulation (EC) No 450/2009, which establishes which active, intelligent, and article materials can enter in contact with food, and Commission Regulation (EC) No 1333/2008, related to food additives [67,68,69].

Edible films and coatings composed of biopolymers and enriched with active compounds have raised the interest of the food industry and technologists for application in fish, meat, and derived products in order to prevent lipid/protein/pigment oxidation, off-odors, off-flavors, moisture and color loss, oxygen penetration into the food matrix, and solute transport out of the food, and, therefore, improve preservation, quality, and sensorial properties of the products [64,67,68]. Besides this, these films/coatings add value to food products, as they increase their shelf-life by reducing/inhibiting the growth of spoilage and pathogenic microorganisms [64,67].

However, edible film and coating technologies have some associated concerns for both consumers (food safety) and the food industry, such as the initial investment and production cost, equipment and production process complexity, scale-up process, and associated regulations [41,64]. Table 6 shows some examples of edible films and coatings enriched with active compounds applied to fishery products and their effects on physicochemical, quality, and sensorial properties and the shelf-life of these products. Overall, the application of edible films and coatings enriched with active compounds in fish and fishery products enhanced or maintained its quality and sensorial properties, due essentially to its inhibitory action on the growth of spoilage and pathogenic microorganisms, throughout the storage period and, therefore, led to an extension of shelf-life. Ojagh et al. reported an extension from 12 days to 16 days (refrigerated storage, 4 °C) in rainbow trout (*Oncorhynchus mykiss*) coated with a film of chitosan and cinnamon oil [69]. Meanwhile, the shelf-life of beluga sturgeon (*Huso huso*) fillets covered with whey protein concentrate coating with 1.5% cinnamon essential oil (stored at 4 °C) was extended by eight days [70].

### 3.2. Hyperbaric Storage: A Novel Methodology for Fish Preservation

High-pressure processing is used as a promising “nonthermal” technique for food preservation that efficiently inactivates the vegetative microorganisms most commonly related to foodborne diseases. High-pressure processing is carried out with intense pressure in the range of 100–1000 MPa, allowing preservation with minimal effect on food taste and nutritional characteristics [78]. One of the advantages of high-pressure processing is that food products are treated instantly, regardless of their shape and size (isostatic principle). The application of elevated pressures (100–600 MPa) can be used for a variety of food processing and preservation applications, including high-pressure freezing and thawing, blanching, pasteurization, and commercial sterilization [79]. Research about the application of high pressure on fish muscles has been mainly focused on three main areas, including the extension of refrigerated/frozen shelf-life [80,81], pressure-induced texturation (gel-forming) [82], and high-pressure freezing/thawing [83]. 

#### 3.2.1. Hyperbaric Storage

Recently, a novel food preservation storage methodology based on storage under moderated pressure (hyperbaric storage, HS, from 25 to 100 MPa) has attracted interest due to its high potential energy savings and shelf-life extension. HS opened the possibility to store food products and other biomaterials above atmospheric pressure (AP, 0.1 MPa) as a possible enhancement of conventional refrigeration storage, increasing shelf-life and food quality. This methodology allows the storage of food under pressure at subzero (ST), low (LT), and room (RT) temperatures, HS/ST being particularly important for solid foods, on which freezing/thawing can cause substantial damages to cellular/tissue structures, leading to textural modifications [84].

The possibility to use HS for food preservation or other biomaterials occurred by chance, after the observation of recovered items from the research submersible Alvin (owned by Woods Hole Oceanographic Institution), which sank about 1540 m during ten months, containing two bottles filled with bouillon and a plastic box containing sandwiches and apples. The environmental conditions at a depth of 1500 m are fairly constant, and are estimated to be 3–4 °C and ≈15 MPa. After being recovered, the sandwiches appeared fresh by taste and smell, and apples showed no sign of obvious deterioration. The pH value of the apples was the same as fresh ones, and the tyrosinase activity was about half that of a fresh apple [85].

#### 3.2.2. Hyperbaric Storage at Subzero and Low Temperatures

Two freshly dressed whole fishes (pollock and cod, from the species *Gadus chalcogrammus* and *G. morhua*, respectively) were stored at 24.1 MPa and 1 °C during 12 and 21 days, respectively. According to an expert panel, both types of fish were assessed to have better sensory attributes than fish samples stored at the same temperature and AP. The ratings by the expert panel corresponded to the rating these fish samples would have received if they had been stored for a shorter period. This means that the pollock/cod samples stored for 12/21 days with HS at 1 °C received ratings typical of pollock/cod samples stored at AP at 1 °C for 6.7/8.2 days [86].

Some authors used HS/ST without freezing to extend fish shelf-life, while avoiding the damages caused by freezing. This advantage led to the first studies in HS/ST applied in fresh fish (Table 7). High-pressure application decreases the freezing and melting point of water to a minimum of −22 °C at 209 MPa [87], as pressure acts in opposition to the volume increase that occurs with the formation of type I ice crystals, resulting in tissue damage [88]. Charm et al. [86] showed that HS/ST (−3 °C and 22.8 MPa) for 36 days reduced microbial growth on cod fillets, while AP samples presented higher microbial loads. These samples were evaluated by an expert panel, and the HS/ST samples showed comparable or better quality than those stored at AP and −3 or −20 °C. These results suggested that HS/ST is a non-freezing storage method that improves the preservation of cod fillets compared to conventional methods of freezing or refrigeration, because HS/ST inhibits microbial growth and enzymatic activity and prevents damage caused by freezing/thawing. Furthermore, enzymatic degradation of nucleic acid-related substances (ATP, ADP, AMP, and IMP) from carp and chicken muscles stored under HS/ST for 50 days (−8 °C and 110 MPa, or −15 °C and 170 MPa) was slightly slower than for storage at −8 °C (AP), while the enzymatic activity was significantly reduced only by freezing at −18 °C (AP) [89].

Recently, several studies were published regarding the use of HS/LT to preserve fresh fish. Cape hake loins, *Merluccius* spp. [91], and Atlantic mackerel, *S. scombrus*, fillets [93] were both stored at 50 MPa/5 °C for 7and 12 days, respectively, and showed almost no changes in microbial load or TVB-N content compared to the initial samples. For HS samples, an increase was found during storage for water content, water holding capacity, shear resistance, and whiteness. After cooking, HS samples presented a weight lost less than half of the control samples, with no differences in whiteness and only moderate differences by sensorial analysis [91,93].

The quality of Atlantic salmon (*S. salar*) was evaluated by HS/LT (40–60 MPa, 5–15 °C) during 10 days, and a slowdown of spoilage microbial growth was observed, while an additional longer storage experiment (50 days) at 60 MPa/10 ºC revealed microbial inactivation (Fidalgo et al., 2019) [94]. Furthermore, the established limit of total volatile base nitrogen was surpassed at 60 MPa/10 °C after 30 days (contrarily to six days at AP/10 °C), but with stable TMA-N content in the former. Formaldehyde and dimethylamine-nitrogen content increased after six days of HS/LT, but only the former progressively increased until the tenth day, indicating a possible formation by the action of enzymatic activity, but also by other chemical reactions. Additionally, HS/LT slightly increased secondary product content from the lipid oxidation, although to a lower extent compared to AP (at the different storage temperatures). This condition of 60 MPa/10 °C also showed no variations in drip loss, water holding capacity, or myofibrillar fragmentation index in Atlantic salmon, with low changes in muscle fibers, visible by scanning electron micrographs [96]. Furthermore, a decrease of resilience (a texture property) and a retention of fresh-like alcohols and aldehydes (not detected in AP samples after 15 days) were observed in these salmon samples stored at 60 MPa/10 °C for 30 days [96]. 

Proteolytic enzymes and muscle proteins of Atlantic salmon (*S. salar*) were studied under HS [95]. Generally, activities of acid phosphatase, cathepsin B and D, and calpains decreased when compared to fresh salmon, with a more pronounced effect of storage temperature of 37 °C, regardless of the pressure condition. However, activity recovery was observed for some enzymes, as the case of cathepsins B and D, and calpains, which showed an increase of residual activity for samples stored at 60 MPa/10 °C and 75 MPa/25 °C after 50 and 25 days, respectively. A pronounced increase of the myofibrillar fragmentation index was observed at 75 MPa (25/37 °C) after 10 days. Otherwise, at 60 MPa/10 °C, a decrease of myofibrillar fragmentation index values was observed after 50 days of storage. For sarcoplasmic proteins, no effect was observed at 60 MPa/10 °C during 30 days of storage, with a slight increase after 50 days. At 75 MPa/25 °C, a decrease of sarcoplasmic protein content was obtained after 10 days, with no further changes during the 25 days of storage [95].

Spoilage and inoculated surrogate pathogenic (*Bacillus subtilis* endospores, *Escherichia coli*, and *Listeria innocua*) microorganisms were monitored during HS using Atlantic salmon. HS/LT inhibited and inactivated the spoilage microorganisms, and *B. subtilis* endospores reached counts below the detection limit after 30 days, verifying a similar reduction for *E. coli* and *L. innocua* counts [98].

#### 3.2.3. Hyperbaric Storage at Room Temperatures

Similarly, the concept of HS/RT arose as an opportunity to preserve food products, namely fresh fish products, with the published works demonstrating a great opportunity to preserve fresh fish. Tilapia fillets (*O. niloticus*) stored under HS/RT (100 MPa/25 °C) over 12 h revealed almost no changes in the microbial load of mesophiles (4.7 log CFU/g) and psychrophiles (4.59 log CFU/g), and a decrease to about 2.0 log CFU/g when stored at 200 MPa/25 °C for 12 h. Tilapia fillets stored at 200 MPa/25 °C for 12 h showed a lower K-value (40%) than samples stored at AP (92%). This result is significant, because a K-value above 60% indicates putrefaction, and only the HS/RT tilapia fillets were below this limit [90].

Recently, HS at 75 MPa caused a reduction in the initial microbial counts of Atlantic salmon, leading to an increase of the microbial shelf-life of at least 25 days, compared to three days of refrigeration, while, at 60 MPa, a microbial growth slowdown was observed, increasing the microbial shelf-life to at least six days. Additionally, besides the maintenance of muscle color during the 25 days, an enhancement of primary and secondary lipid oxidation products was observed, but to a lower extent compared to AP samples [92]. Later, vacuum-packaged fresh Atlantic salmon loins were studied for 30 days under HS/RT conditions, verifying the retention of important physicochemical properties for at least 15 days, such as fatty acids (n-3 polyunsaturated fatty acids) and fresh-like volatile compounds, or lower lipid oxidation and myofibrillar fragmentation index, while refrigeration after five days showed already volatile spoilage-like compounds due to microbial activity [97].

In addition, spoilage and inoculated surrogate pathogenic (*B. subtilis* endospores, *E. coli*, and *L. innocua*) microorganisms were also monitored during HS/RT using Atlantic salmon. HS/RT inhibited and inactivated the spoilage, and inoculated surrogate pathogenic (*B. subtilis* endospores, *E. coli*, and *L. innocua*) microorganisms reached counts below the detection limit after 30 days, showing that, besides shelf-life extension (due to microbial growth inhibition), it also increased microbial safety (by microbial inactivation) of vacuum-packaged Atlantic salmon [98].

**Figure 2:**
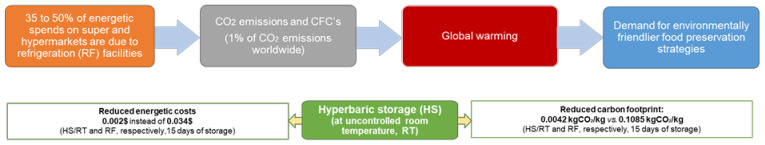
Schematic representation of the energetic costs and environmental impact of hyperbaric storage at uncontrolled room temperature compared to conventional refrigeration [99,100].

This concept of HS/RT arose as an opportunity to preserve food products, providing an opportunity to reduce energy consumption, carbon footprint, and its associated costs (Figure 2). 

**Figure 3:**
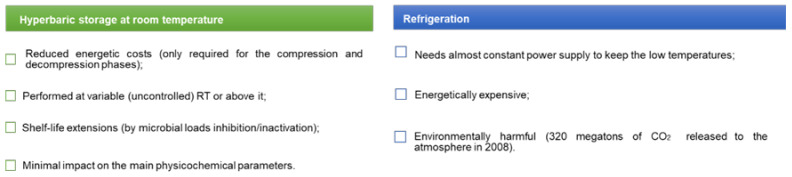
Schematic representation of the advantages of hyperbaric storage at room temperature compared to conventional refrigeration [102].

Consequently, this method has attracted the attention of many researchers during the last few years, and some studies have been made recently to assess the feasibility of this technology for food preservation compared to refrigeration [101]. HS/RT has been shown to inhibit microbial growth at 50–100 MPa, to inactivate microorganisms at higher pressures (100–220 MPa), and to attenuate some of the physicochemical changes that occur during storage at AP, thereby yielding similar or better products than those obtained with refrigerated storage. HS/RT requires energy only during the short compression and decompression phases, and no additional energy is required to maintain the product while stored under pressure for prolonged periods. Energy cost savings with HS/RT were 101investigated by [99], who concluded that energetic costs of HS/RT were lower than refrigerated storage (Figure 3). Additionally, the carbon footprint associated to HS/RT is also lower than refrigeration. With regard to refrigeration, the two main sources of CO_2_ production are from energy utilization and the leakage of cooling gas, while, for HS/RT, the CO_2_ produced by energy consumption is negligible, and the main source of CO_2_ emissions are attributable to the production of construction materials used for the hyperbaric chamber, thereby demonstrating that HS/RT generates considerably less CO_2_ than conventional refrigeration processes [99].

## 4. Conclusions

As stated, fish is a highly perishable food characterized by a short shelf-life. Refrigeration is probably one of the most used methods for fish preservation, along with freezing, and, more recently, superchilling. However, several deteriorative fish quality changes occur during refrigerated storage, particularly in texture, color, and flavor, limiting shelf-life. Frozen storage can avoid these changes (except for texture), but freezing/thawing largely alters the fish fresh-like characteristics. Emerging food packaging techniques, such as the use of edible films and coatings, also meet consumer demands due to their biodegradability and sustainability, while improving the safety and extending the shelf-life of fish and fishery products. Other emergent technologies are arising, as in the case of hyperbaric storage. This methodology uses different pressure and temperature conditions applied at subzero, low, and room temperatures, and has shown the possibility to increase fish shelf-life by microbial inhibition/inactivation, maintaining textural, sensorial, and nutritional characteristics when compared to conventional methods of storage, with the additional advantage of potentially high energy savings, especially when performed at naturally variable/uncontrolled room temperatures. However, currently available high pressure equipment was designed to operate at very high pressure (up to 600 MPa for short minutes), and not to perform hyperbaric storage (up to a maximum of 200 MPa, but for weeks/months), and so specific pressure requirements for hyperbaric storage are of interest to be built. 

## Figures and Tables

**Table 1:** Enzyme action in chilled fish. Adapted from [10].

Enzyme(s)	Substrate(s)	Effect
Glycolytic enzymes	Glycogen	Lactic acid production resulting in pH drop
Nucleotide breakdown enzymes	ATP, ADP, AMP, IMP	Gradual production of Hx
Cathepsins	Proteins, peptides	Softening of tissue
Chymotrypsin, trypsin, carboxypeptidases	Proteins, peptides	Belly-bursting
Calpain	Myofibrillar proteins	Softening of tissue
Collagenases	Connective tissue	Softening and gaping of tissue
TMAO demethylase	TMAO	Formaldehyde production

ATP: Adenosine triphosphatase; ADP: adenosine diphosphate; AMP: adenosine monophosphate; IMP: inosine monophosphate; Hx: hypoxanthine; TMAO: trimethylamine oxide.

**Table 2:** Spoilage compounds produced by microorganisms during the storage of fresh fish. Adapted from [25,26].

Spoilage Bacteria	Spoilage Compound(s) Produced
*Shewanella putrefaciens*	TMA, H_2_S, CH_3_SH, (CH_3_)_2_S, Hx, and acids
*Pseudomonas* spp.	CH_3_SH, (CH_3_)_2_S, ketones, esters, aldehydes, NH_3_, and Hx
*Photobacterium phosphoreum*	TMA and Hx
Vibrionaceae	TMA and H_2_S
Enterobacteriaceae	TMA, H_2_S, ketones, esters, aldehydes, NH_3_, Hx, and acids
Lactic acid bacteria	H_2_S, ketones, esters, aldehydes, NH_3_, and acids
Yeast	Ketones, esters, aldehydes, NH_3_, and acids
Aerobic spoilers	NH_3_, acetic, butyric, and propionic acids
Anaerobic rods	Ketones, esters, aldehydes, and NH_3_

TMA: trimethylamine; H_2_S: hydrogen sulphide; CH_3_SH: methylmercarptan; (CH_3_)_2_S: dimethylsulphide; Hx: hypoxanthine; NH_3_: ammonia.

**Table 3:** Shelf-life of different fish species stored in ice. Adapted from [31,35].

Fish Species	Temperate Waters (Days)	Tropical Waters (Days)
Marine Species	2–24	6–35
Cod (*Gadus morhua*)	9–15	na
Hake (*Merluccius merluccius*)	7–15	na
Catfish	Na	16–19
Batfih (*Ogcocephalus darwini*)	Na	21–24
Halibut (*Hippoglossus stenolepis*)	21–24	na
Sardine (*Sardina pilchardus*)	3–8	9–16
Freshwater species	9–17	6–40
Catfish (*Silurus glanis*)	12–13	15–27
Trout (*Oncorhynchus mykiss*)	9–11	16–24
Perch (*Perca* spp.)	8–17	13–32
Tilapia (*Oreochromis niloticus*)	Na	10–27
Corvina (*Argyrosomus regius*)	Na	30

na—not applicable.

**Table 4:** The effect of superchilling, chilling, and/or freezing technologies and/or the synergistic effect with packaging (vacuum packaging or MAP: modified atmosphere packaging) on the quality and shelf-life of fish muscle foods.

Species (Scientific Name)	Storage Conditions	Main Results	References
Atlantic Salmon (*Salmo salar*)	Superchilling: −1.0 °C up to 16 days Chilling: +4.0 °C up to 16 days Freezing: −40.0 °C up to 30 days	Superchilled salmon for nine days, without salting, presented the best results when compared with ice-chilled and frozen samples due to the reduction of biochemical quality degradation and a low/less degree of protein denaturation and structural damage. The highest production yield of salted salmon (in the weight of the salmon) was observed for the superchilling method at the ninth day of storage.	[45]
	Superchilling: −1.4 °C and −3.6 °C up to 34 days Chilling: +4.0 °C up to 21 days Freezing: −40.0 °C up to 37 days Packaging: Vacuum	The storage time of vacuum-packed, superchilled salmon fillets can be doubled when compared to ice-chilled samples. The highest drip loss value and degree of protein and myosin denaturation for superchilled salmon stored at −1.4 °C. Better muscle hardness in superchilled samples stored at −3.6 °C and stable activity of cathepsins enzymes (B and B + L) in all salmon samples.	[46]
	Superchilling: −1.5 °C up to 28 days Chilling: +2.0 °C up to 21 days Packaging: MAP-CO_2_, N_2_ (CO_2_ compositions: 25%, 40%, 60%, 75%, and 90% with different gas-to-product ratios).	Extension of shelf life for superchilled product (using 90% CO_2_) salmon fillets, from 11 days to 22 days, compared to chilled/control samples.	[44]
	Superchilling: −2.0 °C air overwrap up to 24 days Chilling: +4.0 °C air overwrap up to 24 days Packaging: MAP—60% CO_2_, 40% N_2_	Spoilage of MAP and air-stored salmon fillets after 10 days and seven days, respectively. Good microbial and sensorial quality during 24 days of storage for superchilled MAP salmon, compared to 21 days of superchilled salmon with air overwrap.	[47]
	Superchilling: −1.5 °C up to 28 days Packaging: Vacuum	The liquid loss value of the superchilled salmon fillets was significant after one day of storage and not significant between three and 14 days of storage, and after 21 days, this parameter decreased. The drip loss parameter of the superchilled salmon fillets remained without significant differences between one and 14 days of storage, but increased after 21 days.	[48]
Cod (*Gadus morhua*)	Superchilling: −1.5 °C up to 15 days Chilling: +0.5 °C up to 15 days	The shelf-life of superchilled cod fillets increased by three days when compared to the chilling process, resulting in a total of 15 days of shelf-life. The shelf-life of cod samples stored at a chilled temperature (+0.5 °C) only increased from 12.5 to 14 days.	[49]
	Superchilling: −1.0 °C up to 42 days Chilling: +4.0 °C up to 37 days Freezing: −21.0 °C for 36 days or −40.0 °C for 43 days Packaging: Vacuum	The superchilling technology combined with vacuum packaging prolonged the shelf-life of the cod fillets by several weeks when compared to the traditional chilling method. Drip loss and liquid loss parameters of superchilled cod fillets decreased and increased, respectively, compared to the chilled samples.	[38]
	Superchilling: −0.9 °C up to 21 days Chilling: +1.5 °C up to 21 days Packaging: MAP—50% CO_2_, 45% N_2,_ 5% O_2_	The shelf-life of fresh cod loins has been extended from nine days for ice-chilled storage to 16 or 17 days by superchilled storage. In addition, MAP combined with chilling and superchilling methods increased the shelf-life to 14 and 21 days, respectively. The superchilled MAP cod loins after seven days showed significant differences in muscle texture when compared to other storage methods due to the formation of ice crystals, when the storage temperature reached the freezing point of the fish.	[43]
	Superchilling: −2.0 °C or −3.6 °C up to four weeks Chilling: +0.0 °C up to four weeks Packaging: MAP—50% CO_2_, 45% N_2,_ 5% O_2_	The effect of brine (2.5 ± 1.0% NaCl) on cod loins was evaluated using the combined superchilling/MAP technology. The synergistic effect of superchilling/MAP extended the shelf-life of unbrined cod loins by 21 days (at −2.0 °C) instead of 14 to 15 days (at −2.0 °C) of the superchilled samples. The brined samples showed a shorter shelf-life compared to unbrined samples, especially in superchilling/MAP cod loins.	[50]
Tilapia (*Oreochromis niloticus*) fillets	Superchilling: −1.0 °C Chilling: +1.0 °C Packaging: MAP—50% CO_2,_ 50% N_2_	The MAP tilapia fillets remained good for consumption at the microbiological level, even after 23 days of storage at both temperatures (−1.0 °C and +1.0 °C). However, some detrimental effects were observed in color, drip loss, and texture of samples. The best storage conditions, considering both sensorial evaluation and microbial counts, were 13–15 and 21 days for the chilled and superchilled tilapia fillets, respectively.	[51]

**Table 5:** Shelf-life of fish species stored at different freezing temperatures. Adapted from [56].

Type of Fish	Storage Time (Months)		
	−18 °C	−25 °C	−30 °C
Fatty fish (sardines, salmon)	4	8	12
Lean fish (cod, haddock)	8	18	24
Flat fish (flounder, plaice)	9	18	24

**Table 6:** Edible film and coating enriched with active compounds applied for fishery products.

Fish Product	Film/Coating Material	Active Compounds	Main Results	Reference
Beluga Sturegeon (*Huso huso*) fillet	Coating composed of 8.0% (*w*/*v*) whey protein concentrate, glycerol, and Tween 80	Cinnamon essential oil (1.5% *v*/*v*)	Shelf-life extension by eight days (compared to uncoated control samples) by reducing lipid oxidation and microbial activity.	[70]
Bluefin tuna (*Thunnus thynnus*) slides	Film composed of 1.0% (*w*/*v*) fish myofibrillar protein, 25.0% (*w*/*w*) glycerol, and 2.0% (*w*/*w*) MTGase	Catechin-Kradon (*Careya sphaerica* Roxb.) extract (0.9% *v*/*v*)	Maintenance of sensorial qualities after eight days in comparison to the four days of the unwrapped control samples, due to the reduced microbial growth, lipid oxidation, and formation of metmyoglobin.	[71]
Freshwater rainbow trout (*Oncorhynchus mykiss*) fillets	Coating composed of 1.0% (*w*/*w*) carrageenan	Essential lemon oil (1.0% *w*/*w*)	The lemon essential oil incorporated in the carrageenan coating and applied to samples of rainbow trout fillets showed good antimicrobial activity and reduced lipid oxidation during the 15 days of storage at 4 °C.	[72]
Grass carp (*Ctenopharyngodon idellus*) fillets	Coating composed of 2.0% (*w*/*v*) chitosan	Glycerol monolaurate (0.1% and 0.3%)	After 20 days of storage at refrigerated temperature (4 °C), grass carp fillets maintained microbial safety, good quality, and sensorial properties.	[73]
Rainbow trout (*Oncorhynchus mykiss*)	Coating composed of 2.0% (*w*/*v*) chitosan, 1.0% acetic acid, 0.75% glycerol, and 0.2% Tween 80	Cinnamon oil (1.5% *v*/*v*)	The coated rainbow trout during the refrigerated storage (4 °C) for 16 days maintained the overall quality and sensorial properties without significant microbial growth, compared to 12 days of shelf-life for the control samples.	[69]
Rainbow trout (*Oncorhynchus mykiss*) fillets	Film composed of 1.5% (*w*/*w*) quince seed mucilage (QSM), 35.0% (*w*/*w*) glycerol, and 0.1–0.2% (*w*/*v*) Tween 80	Oregano or thyme essential oils (1.0, 1.5, or 2.0% *v*/*v*)	Shelf-life extension of rainbow trout fillets of up to 11 days compared to the control samples.	[74]
Rainbow trout (*Oncorhynchus mykiss*) slices	Film composed of 75.0% fish gelatin and 25.0% sodium alginate, glycerol, and Tween 80	Oregano essential oil (OEO) (1.5% *w*/*v*)	The use of OEO gelatin-alginate film decreased microbial growth during the 15 days of the storage period compared to the control samples.	[75]
Red drum (*Sciaenops ocellatus*) fillets	Coating composed of 1.5% (*w*/*v*) chitosan, 25% glycerol, and 1.0% (*v*/*v*) acetic acid	Grape seed extract (0.2% *w*/*v*) or tea polyphenols (0.2% *w*/*v*)	Considering the results of microbiological, physicochemical (such as pH, TVB-N value, etc.), and sensorial analysis of red drum fillet samples immersed in the active coating, the shelf-life was extended by six to eight days in refrigerated storage (4 °C) when compared to the uncoated control samples.	[76]
Salmon slices *	Film composed of 8.0% (*w*/*v*) catfish-skin gelatin and chitosan, sorbitol, and glycerol	Clove essential oil (7.5% *v/w*)	Samples of salmon slices wrapped in edible films of gelatin-chitosan with clove essential oil showed an inhibitory action on spoilage and pathogenic microorganisms when stored at 2 °C, with a shelf-life of 11 days against nine days of the control samples.	[77]

* The authors did not mention the scientific name of the species.

**Table 7:** Main results of the application of hyperbaric storage (HS) on fresh fish.

Samples	Storage Conditions	Main Results	References
Cod fish fillets (*Gadus morhua*)	22.8 MPa, at −3 °C for 36 days	An expert panel rated these samples with a similar or better quality than samples stored at −3 °C and at atmospheric pressure.	[86]
Dressed pollock (*Pollachius pollachius*)	24.1 MPa, at 1 °C for 12 days	Microbial load with no changes during storage time.	[86]
Dressed cod fish (*Gadus morhua*)	24.1 MPa, at 1 °C for 21 days		
Carp *	110 MPa/−8 °C and 170 MPa/−15 °C for 50 days	Enzymatic activity associated to nucleic acid degradation was slower than in samples stored at −8 °C and at atmospheric pressure.	[89]
Tilapia fillets (*Oreochromis niloticus*)	100–200 MPa, at 25 °C for 12 h	Total plate counts remained stable at 100 MPa and showed a reduction of about two log units at 200 MPa.	[90]
Cape hake loins (*Merluccius capensis*)	50 MPa, at 5 °C for seven days	Microbial counts and total volatile basic nitrogen content remained unaltered during storage. Drip losses, shear resistance, and whiteness increased, but, after cooking, these differences almost disappeared.	[91]
Atlantic salmon (*Salmo salar*)	50–75 MPa, at 25–37 °C for 10 days	Initial microbial counts were reduced at 75 MPa, while no effect was observed at 50 MPa, and there was an inhibition at 60 MPa. No effect on color parameters. Increase of lipid oxidation state compared to refrigeration.	[92]
Atlantic mackerel (*Scomber scombrus*, L.) fillets	50 MPa, at 5 °C for 12 days	Initial microbial counts were maintained or reduced. No significant lipid degradation was observed, and better fish quality indicators were observed (pH, TVB-N, drip loss, water holding capacity, and firmness after cooking) than under refrigeration.	[93]
Atlantic salmon (*Salmo salar*)	40–60 MPa at 5–15 °C for 10 days	Microbial growth was slowed down with inactivation at 60 MPa. Low values of volatile base nitrogen at 60 MPa up to 15 days with stable trimethylamine-nitrogen. Increase of formaldehyde and dimethylamine-nitrogen content.	[94]
	50–75 MPa at 10–37 °C for 10 days	Initial activities of acid phosphatase, cathepsin B and D, and calpains decreased by increasing the storage temperature. A pronounced increase of myofibrillar fragmentation index at 75 MPa and 25 or 37 °C after 10 days.	[95]
	60 MPa at 10 °C for 30 days	No variations in drip loss, water holding capacity, or myofibrillar fragmentation index. Low changes in muscle fibers, visible by scanning electron micrographs, and a decrease of the resilience property. No effect on fatty acids content, with a lower polyene index compared to refrigeration. Retention of fresh-like volatile compounds.	[96]
	75 MPa at 25 °C for 30 days	Only resilience (textural property) was affected, decreasing after 30 days. Slower myofibrillar fragmentation index and no effect on fatty acids content, with a lower polyene index, compared to refrigeration. Retention of fresh-like volatile compounds.	[97]
	60 MPa/10 °C and 75 MPa/25 °C for 30 days	Inhibition and inactivation the spoilage microorganisms. Reduction of surrogate pathogenic microorganism (*Bacillus subtilis* endospores, *Escherichia coli*, and *Listeria innocua*) counts.	[98]

* The authors did not mention the scientific name of the species.

## Data Availability

The data that support the findings of this study are available from the corresponding author, J.A.S., upon reasonable request.
